# Community-based (midstream) approaches to improving child oral health across the UK

**DOI:** 10.1038/s41415-026-0045-5

**Published:** 2026-11-13

**Authors:** Declan Cairns, Zoe Marshman, Laura Crawford, Michael Donaldson, Mary Wilson, Stefan Serban

**Affiliations:** 41415877387001 NHS Forth Valley, NHS Grampian, UK; 41415877387002 School of Clinical Dentistry, University of Sheffield, Sheffield, UK; 41415877387003 Department of Health (Northern Ireland), Northern Ireland, UK; 41415877387004 Public Health Wales, Wales, UK; 41415877387005 School of Medicine, Dentistry, and Nursing, University of Glasgow, Glasgow, UK

## Abstract

As health is a devolved matter in the UK, each of the four countries has developed a unique approach to improving the oral health of children in order to meet the needs of their population. Public health interventions can be classed into three main categories: national level (upstream) policy initiatives, community-based (midstream) interventions, and individual level (downstream) interventions. Midstream interventions aimed at improving child oral health have been implemented to varying degrees across all four countries of the UK. This article presents a descriptive overview of the main midstream child oral health improvement programmes in each of these four countries, identifying key challenges and opportunities presented around each approach and the policy framework in which they are operating. Common facilitators across all four countries included cross-party political support and integration with early years services, while sustainability of funding and workforce capacity were identified as challenges to implementation. Robust long-term monitoring and evaluation frameworks are needed to inform effective resource allocation and sustain the improvements achieved.

## Key points

- Various community-based interventions including supervised toothbrushing programmes are delivered across the UK, reflecting the strength of the evidence.
- Facilitators include cross-party political support and integration with early-years services. Barriers include funding sustainability and workforce pressures.
- Midstream programmes play an important role in oral health improvement alongside upstream action. There is a need for robust long-term monitoring and evaluation.

## Introduction

Despite improvements in oral health over the past few decades, childhood caries remains a major public health challenge.^1^ Children from the most socioeconomically disadvantaged backgrounds are most affected by this condition both in terms of prevalence and severity.^2^ Dental extractions continue to be one of the leading causes of hospital admission for children aged 5–9 years, with those living in the most deprived areas being more than three times more likely to require this intervention than those living in less deprived areas.^3^

Health has been a devolved matter within the four countries of the UK since 1999, and each nation has developed a different approach for oral health improvement for children to meet the needs of their populations. A well-established model for public health interventions is described within the upstream (policy), downstream (individual level) and midstream (community) framework.^4^ As such, ‘upstream interventions' are interventions at national policy level such as the Soft Drinks Industry Levy or Minimum Unit Pricing for Alcohol.^5^ These interventions aim to reduce consumption and related harms, particularly among socioeconomically disadvantaged groups. ‘Downstream' interventions are focused on interventions delivered at individual level, such as chairside clinical care. Midstream interventions focus on community-level support, seeking to empower individuals to engage in health-promoting behaviours, and targeting ‘at-risk' groups. Midstream interventions are often used to reduce social inequalities in caries experience with different approaches to targeting used. Such interventions are very relevant for children as they can be delivered via existing child health programmes or education settings.^6^ They rely on formal and informal partnership working with communities and local agencies supporting children including health visitors, nursery and school staff, and the voluntary sector, in order to embed oral health support within settings that families are already engaging with.^7^

This article aims to provide an overview of midstream (community level) interventions for child oral health improvement currently delivered across the four countries of the UK and explore some of the barriers and facilitators for implementing these interventions.

## Methods

This article presents a descriptive overview of midstream community-based child oral health improvement programmes across the four countries of the UK. The section for each country was authored by the national lead or other experts responsible for child oral health improvement programmes in their respective country. A structured descriptive framework was applied across all four nations, capturing: target population, delivery mechanisms, commissioning and funding models, workforce, monitoring and evaluation approaches, and key barriers and facilitators for implementation. This framework was used to support a structured overview and to ensure consistency of reporting. This overview focuses on programmes delivered at national level; locally commissioned interventions, where considerable variation exists, are not captured in this review.

## Results

The following section provides an overview of the main community-based child oral health improvement programmes in each of the four countries of the UK in alphabetical order. A brief summary overview of these can be found in Table 1.

**Table 1:** Community based child oral health improvement programmes across the UK

**Intervention**	**England**	**Northern Ireland**	**Scotland**	**Wales**
Oral health support for families through health visiting teams	✔	✔	✔	✔
	Universal	Universal	Universal	Universal
Delivery of OH Packs (toothbrush/toothpaste)	Variable	✔	✔	✔
	Targeted	Targeted	Universal	Universal
Supervised toothbrushing nurseries	✔	✔	✔	✔
	Targeted	Targeted	Universal	Targeted
Supervised toothbrushing schools	✔	Commencing 2025/26	✔	✔
	Targeted	Targeted	Targeted	Targeted
Healthy eating policies for nurseries and schools	Varies across local authorities	✔	Supported by Childsmile but not delivered directly through the programme	Supported by D2S but not delivered directly through the programme
	Varied	Universal and targeted	Universal	Universal
Community engagement^†^	Varies across country	X	✔	Opportunistic events and partnerships
	Targeted	X	Targeted	Targeted
D2S, Designed to Smile; OH, oral health. ^†^Community engagement in this context is defined as activities aimed at addressing oral health inequalities through engaging with disadvantaged communities and offering oral health support to families with young children.				

### England

Local authorities (LAs) bear statutory responsibility for oral health improvement in England under the Health and Social Care Act 2012, leading to variations in the interventions commissioned in line with local arrangements and priorities. In some LAs, specific child oral health improvement programmes are commissioned while in others a more generic approach is taken as part of improving children's health more generally. While there is no distinct national oral health improvement programme for children, several key midstream interventions are delivered across the country. These include, but are not limited to:

#### Health visiting teams offering oral health support

The Healthy Child Programme in England includes the promotion of oral health.^8^ Mandated reviews by health visiting (HV) during early childhood (ages 0–3 years) offer structured opportunities to deliver oral health support and guidance to parents. However, there is wide variation between LAs in HV training, the level of oral health support incorporated into these visits, and whether they are able to offer toothbrushing packs. Examples of different approaches that have been found to be acceptable to those involved include: 1) the First Dental Steps programme in the South West of England^9^ with a focus on training, distribution of packs, a referral pathway for high-risk children into the Community Dental Service and a data capture template for HV teams;^10^ and 2) the HABIT intervention which includes HV training, an online resource for parents and a paper-based leaflet with an action plan.^11^

#### Supervised toothbrushing programmes

Supervised toothbrushing programmes (STP) are also delivered in early years settings, for children aged 3–6 years old. STP are generally targeted at the 20% most deprived areas; however, the coverage of each varies between LAs. There has been an increase in the number of STPs implemented across the country over the last three years with 240,000 children taking part in May 2025. The Department of Health and Social Care announced funding for four years to roll out STP to 600,000 children in England. The recent BRUSH study^12^ highlighted several barriers and facilitators to delivering supervised toothbrushing, with uncertain funding, logistical challenges, and competing priorities being noted as the most significant challenges to implementation. However, there has been a broad recognition among policymakers, teaching staff and parents about the benefits of STP for reducing dental caries among children, as well as highlighting the benefits of giving individual schools and nurseries the flexibility to tailor the timing, delivery methods and resources to fit the needs of their setting for improving engagement. Due to the variation between LAs and different configuration of STPs, evaluation of the effectiveness is challenging particularly investigating whether the children participating are those with the most potential to benefit.

### Northern Ireland

The ‘Happy Smiles' programme is a national initiative in Northern Ireland (NI) designed to improve the oral health of children aged 3–4 years old in education authority funded pre-schools.

The Department of Health in NI funds the programme, with the Strategic Planning and Performance Group identifying the areas of greatest need and commissioning the services as appropriate. Funding is then provided to each Health and Social Care Trust (HSCT), where oral health improvement teams deliver the programme.

Happy Smiles is a targeted midstream intervention, aimed at the 20% most deprived pre-school facilities in each of the HSCTs. The programme has three main elements:

- The ‘Happy Smiles Supervised Toothbrushing Programme' delivers daily supervised toothbrushing in pre-school establishments
- ‘Happy Smiles Healthy Snacks' aims to reduce the frequency of sugar intake and increase access to fruits by introducing a written break-time food policy for participating facilities, which forms part of the overall school health policy
- ‘Happy Smiles Education Programme' promotes prevention through education on diet and regular dental attendance.

The regional development of resources that can be implemented across all five health trusts is one of the key strengths of Happy Smiles. Staffing levels and staff availability remain one of the biggest challenges for implementing Happy Smiles, both in HSCT teams and pre-school facilities. There has also been some resistance from some pre-school staff pointing towards parents' responsibilities regarding children's toothbrushing and issues with funding keeping pace with the cost of delivery which have impacted the HSCT's ability to maintain engagement. The challenges associated with recovering from the COVID-19 pandemic continue to have a detrimental effect on recruiting new target facilities into the programme, given the aforementioned challenges.

Despite these challenges, a recent review of the programme found 97 of the 190 target pre-school facilities are currently participating in the programme (51%) with levels of participation varying across each HSCT, there were a further 231 non-target facilities participating. This gives a total of 328 of 735 pre-school facilities participating in the programme (45%).

An evaluation of the Happy Smiles Programme reported improvements in children's oral health evident in the reduction in prevalence and severity of caries in five-year-old children via oral health survey data,^13^^,^^14^ the decrease in extractions and fillings^15^ for children within the general dental service and a decrease in the number of children undergoing general anaesthesia for tooth extraction. Evaluation findings must be interpreted with caution, improvements in oral health cannot be directly attributed to Happy Smiles and may reflect wider system-level changes. There are currently plans to develop regular monitoring and evaluation of the programme.

### Scotland

Childsmile is the national oral health improvement programme for children in Scotland, delivering a range of population-wide universal as well as targeted interventions aimed at children from the most socioeconomically deprived backgrounds.^16^ A combination of policy-driven actions and supportive legislation governs the programme, including the Children and Young People (Scotland) Act 2014^17^ and the Public Health (Scotland) Act 2008.^18^ The programme is funded nationally by the Scottish Government and delivered locally by National Health Service (NHS) Health Boards.

Since the introduction of Childsmile twenty years ago, there has been a significant reduction in the prevalence of dental caries in five-year-old children in Scotland from nearly 60% to 26%.^7^ Through a proportionately universal approach a blend of universal and targeted oral health improvement interventions are delivered to parents/carers and children at key points throughout their development.

The main elements of the programme are:

- Childsmile daily supervised toothbrushing – aimed at all children attending nursery and targeted to the 20% of primary one and two classes situated in areas with the highest level of need
- Oral health packs, containing (1,450 ppmF) toothpaste and toothbrushes, are also distributed universally to children for use at home – procured via a national contract
- Childsmile Community and Practice – offering targeted oral health support to families with young children in the most disadvantaged communities, in the family home. Dental health support workers engage children and their parents/carers from birth, helping facilitate attendance at the dentist and linking families with community groups and organisations that can help them for wider support
- Primary dental care services – deliver both universal and targeted components of Childsmile, including toothbrushing instruction/demonstration, dietary advice, and fluoride varnish application
- School-based fluoride varnish application – applied twice per year to the children in the 20% most deprived schools.

A significant strength of the programme is its integration with other services, including early years health visiting services, the education sector, and with wider non-healthcare settings. This multi-sector approach enabled the programme to be embedded within non-clinical settings enhancing the focus on prevention rather than reactive treatment. Another key strength of Childsmile is the continuous monitoring and evaluation which provides robust evidence base for improving the effectiveness of operational delivery of the programme.^19^ As a result, Childsmile has been recognised by the European Commission as a best practice public health intervention and elements of the programme have informed the development of child oral health improvement initiatives internationally, including in Chile, Malawi, Romania, and beyond.^20^^,^^21^^,^^22^

Despite these successes, inequalities remain between children from the most and the least socioeconomically deprived areas. The overall prevalence of caries has improved across all deprivation groups but the gradient between groups continues to remain a challenge.^23^ A key strength of the programme is cross-party support across successive Scottish Governments. Public health interventions often produce population wide results only after several years of continuous implementation, making them vulnerable to changes in political cycles. This sustained political commitment has allowed Childsmile to deliver long term improvements in oral health outcomes at a population level.^5^

The programme activity is monitored and evaluated by the University of Glasgow and Public Health Scotland using a bespoke national IT system (developed and managed by the University of Dundee, Health Informatics Centre). Additionally, child oral health epidemiological data are recorded annually through the National Dental Inspection Programme.

### Wales

Designed to Smile (D2S) is the national oral health improvement programme for children in Wales, from birth to around seven years old. The programme employs a combination of targeted and universal interventions to reduce the prevalence of dental caries and narrow socio-economic inequalities in oral health across the country. Oral health improvement is primarily funded by the Welsh Government and delivered through the D2S programme. D2S has a ring-fenced budget, sits within NHS Wales Community Dental Services and is strategically led by the Dental Public Health team in Public Health Wales. The programme works collaboratively with early years services in local health boards, education and social care, and several other key stakeholders.

The universal component of D2S focuses on prevention for children from birth by establishing good habits early, providing advice to families with young children, supplying toothbrushing packs, and facilitating pathways into clinical dental services. This intervention is integrated into the operating model of the national Healthy Child Wales programme,^24^ with a nationally consistent approach to training of health visitors and other professionals by Designed to Smile staff.

Like the other nations, D2S also delivers a supervised toothbrushing programme, aimed at nurseries and primary schools situated in more disadvantaged areas (approximately 60% of settings in Wales are targeted), and accompanied by regular distribution of toothbrushing home packs.

One of the key successes of D2S is its cross-party support for the programme and the development of a national identity that supports partnership working across teams in NHS Wales and Local Authorities. A committed and enthusiastic workforce supports this, and the longevity and success of the programme have led to it being embedded within the environments in which it acts.

In line with other nations, funding for the programme has struggled to keep pace with the inflationary costs of delivery. Similarly, the hiatus of the programme caused by the COVID-19 pandemic has presented challenges for establishments previously committed to D2S, as well as difficulties in engaging with new institutions.

Dental epidemiology surveys in Wales report a reduction in the prevalence of dental caries; however, some surveys did not attempt to compare schools participating in D2S with those that were not, as they are subject to several factors such as the length of time participating, the level of commitment to D2S and the movement of children from nursery to school. Output measures for D2S are reported annually, including participation rates, consent rates, activity, and the number of resources distributed. Insight work into the barriers and facilitators of participation is currently being conducted by the Dental Public Health Unit at Cardiff University. There is ambition to implement any recommendations that may minimise the barriers to participation through both national work and local initiatives. Sustaining capacity within the national leadership team and our research partner is necessary to ensure vital evaluation activity is an integral part of Designed to Smile.

## Discussion

Over the last decades there have been varying degrees of improvement in children's oral health across each of the four countries of the UK. Oral health inequalities between children living in the most and the least socioeconomically deprived areas are still persistent across all four countries.^25^ While overall improvements in oral health should be rightly celebrated, there is scope to go further to reduce the levels of inequalities. To address this, a combination of both targeted and universal interventions may need to be considered, using the principles of proportionate universalism. This public health approach suggests the delivery of universal interventions for everyone but with the scale and intensity of the intervention proportionate with the level of disadvantage along the social gradient.^23^^,^^26^ The effectiveness of midstream public health interventions at reducing inequalities across the social gradient needs to be considered against the sustained health eroding effects driven by the social, commercial, and political determinants of health.^5^ These structural forces affect disproportionately children from the most socioeconomically deprived backgrounds and act as a counterforce to the gains achieved through community based public health programmes. Upstream policy action is therefore required to address not only the health consequences of these determinants, but the determinants themselves.

Supervised toothbrushing programmes have been implemented in each of the four countries of the UK. These were started in different times and are supported by different commissioning arrangements and delivery models, with a combination of targeted and universal approaches. For some children participation in these programmes may be the only opportunity to use fluoride toothpaste and receive the protection required to reduce their risk of dental caries. In addition, this consistent reinforcement of positive oral health behaviours from an early age can be carried through into later life.

Factors such as political support, monitoring and evaluation, and integration with early years services have been effective in facilitating the implementation of midstream interventions across all four countries. However, issues around funding sustainability and workforce capacity present challenges. While the key lessons on approaches to improving children's oral health are transferable across countries, there is a risk that over-reliance on midstream interventions will be ineffective without suitable complementary upstream policy action.

This paper draws on the expertise of national leads and experts responsible for child oral health improvement programmes in each of the four countries, providing contextual insight that would not be achievable through a literature search alone. At the same time, this may have produced some reporting bias. To mitigate this, all authors have critically reviewed the overall manuscript, not just their own section. The use of a structured descriptive framework applied across all four countries supports consistency and robustness. This overview focuses on interventions delivered at national level, we recognise that there may be locally commissioned programmes with positive outcomes, but these fall outside the scope of this work.

Further research may need to consider the equity of reach of these programmes and the extent to which they are consistently engaging children from the most socioeconomically disadvantaged backgrounds. Additionally, robust evidence around the most effective combinations of upstream, midstream and downstream interventions for reducing inequalities along the social gradient is needed.

## Conclusion

Midstream interventions to improve oral health have been implemented to varying degrees across all four countries of the UK. Despite variations in delivery models, supervised toothbrushing programmes have been adopted in each nation, reflecting the strength of the evidence base for this intervention.

The principles of proportionate universalism were adopted across most settings, combining whole-population (universal) and targeted delivery proportionate with the level of need. Midstream interventions should not be considered in isolation, their success depends on consistent upstream policy decisions and effective downstream chairside interventions, and sustained investment across all three levels is essential. The development of robust evaluation frameworks is essential to generate the evidence needed to inform effective and sustainable resource allocations across upstream, midstream and downstream interventions.
